# Detection and Location of Defects in Externally Bonded FRP Concrete Structures—Comparison of Selected Methods

**DOI:** 10.3390/ma18225090

**Published:** 2025-11-09

**Authors:** Paweł Tworzewski, Kamil Bacharz, Wiktor Wciślik, Michał Teodorczyk, Sylwia Wciślik, Justyna Tworzewska

**Affiliations:** 1 Department of Building Structures, Kielce University of Technology, al. Tysiąclecia Państwa Polskiego 7, 25-314 Kielce, Poland; j.tworzewska@tu.kielce.pl; 2 Department of Strength of Materials and Structures Diagnostics, Kielce University of Technology, al. Tysiąclecia Państwa Polskiego 7, 25-314 Kielce, Poland; kbacharz@tu.kielce.pl (K.B.); wwcislik@tu.kielce.pl (W.W.); 3 Building Structures, Geotechnics and Concrete Department, Instytut Techniki Budowlanej, ul. Filtrowa 1, 00-611 Warsaw, Poland; 4 Faculty of Environmental Engineering, Geomatics and Renewable Energy, Kielce University of Technology, al. Tysiąclecia Państwa Polskiego 7, 25-314 Kielce, Poland; sylwiazw@tu.kielce.pl

**Keywords:** nondestructive testing (NDT), Externally Bonded FRP (EBR), structural health monitoring (SHM), ground penetrating radar (GPR), infrared thermography (IRT), acoustic wave velocity (WVMM)

## Abstract

This paper compares three nondestructive methods used to detect and locate defects such as delaminations or voids in externally bonded fiber reinforced polymer (FRP) concrete structures: infrared thermography, ground-penetrating radar, and measurement of acoustic wave velocity. One of the main goals was to check whether it was possible to distinguish overlapping defects. For this purpose, eight concrete samples were made with a bonded carbon fiber reinforced polymer (CFRP) strip with the following dimensions 100 × 100 × 500 mm. Two samples had no defects, four had single defects varying in location (at the edge of the strip or in the centre) simulating delamination or voids in the concrete cover, and the remaining samples had overlapping defects. Both infrared thermography and acoustic wave velocity measurement methods allow the detection of defects/voids in the adhesive layer and a concrete defect (void in the concrete cover). However, ground penetration failed to detect defects in the adhesive layer. Only infrared thermography allows for the differentiation of overlapping defects. On the basis of the conducted research, the methodology, differences, advantages, and limitations of each method were described, along with recommendations based on the authors’ experience.

## 1. Introduction

Carbon fibre-reinforced polymers (CFRP) are widely used in various industries, including aviation, transportation, and construction, because of their exceptional characteristics such as low weight, high mechanical strength, and resistance to corrosion. In the CFRP construction sector, the application is steadily increasing, with projected usage of 6.2 kilotons by 2025 [1]. In particular, approximately 80–90% of CFRP employed in this field is dedicated to strengthening and rehabilitating existing structures that have suffered deterioration. It is important to emphasise that the number of buildings requiring strengthening is constantly growing. This is due to the natural ageing of building materials, increased service loads, and changes in the standards requirements. New buildings also require strengthening due to changing investor requirements, often already in the construction stage [2].

Materials such as FRP plates, strips, or sheets are commonly used to strengthen RC structures and are among the most effective solutions. However, this solution still has certain limitations related to fully utilising the potential of FRP materials. This is, of course, the issue of debonding, which limits the effectiveness of these solutions and is the most common cause of failure. The causes and factors that initiate debonding are varied, but the main ones include [3,4,5,6]:

- Debonding caused by shear cracks or flexural cracks, i.e., defects in the reinforced concrete structure;
- Debonding related to insufficient anchoring of the FRP material;
- Debonding caused by unevenness or other defects in the concrete surface;
- Delamination in FRP;
- Debonding caused by improper preparation of the concrete surface before applying the FRP material or by improper application.

It should be emphasised that the above-mentioned factors mentioned above are of different significance depending on the strengthening system used. Systems made with externally bonded reinforcement (EBR) are most susceptible to these factors. Near-surface mounted reinforcement (NSM), due to its different application method, excludes or limits the impact of some of the factors mentioned above, which does not eliminate the possibility of debonding, but only limits it [7,8].

The surface to which the FRP EBR is bonded should be level, as concave surfaces can result in FRP debonding. Requirements for the depth of surface variation with respect to a straight base length depend on the type of FRP EBR used. Example requirements [9] are presented in Table 1.

All of the requirements listed above are intended to eliminate or limit potential defects that could lead to premature debonding of the FRP material and failure to achieve the required load-bearing capacity.

Detection and localization of potential FRP debonding initiation point is crucial in the diagnostics and monitoring of objects [3,10,11]. These mainly concern concrete defects (cracks and voids), bond defects (debonding in the adhesive, debonding at the interfaces between concrete and adhesive or adhesive and FRP), and defects in the FRP composite itself, whether prefab type or wet lay-up type.

In this paper, the authors verify the possibility of using selected methods, i.e., infrared thermography, ground-penetrating radar, and measurement of acoustic wave velocity, to locate and determine various types of defects such as voids in the adhesive layer and voids in the concrete cover for externally bound FRP Concrete Structures.

This work not only tested the ability to detect various types of defects, as has already been presented in other works, but also examined whether it was possible to distinguish overlapping defects. In this case, it was a defect/void in the adhesive layer and a concrete defect (void in the concrete cover). It should be noted that such a configuration occurs quite often in the case of FRP deponding. Typical triggers for this phenomenon are cracks or other defects, such as voids in the concrete cover, concave surfaces that lead to the debonding of the FRP material around it.

### 1.1. Infrared Thermography (IRT)—Description of the Method

**Figure 1:**
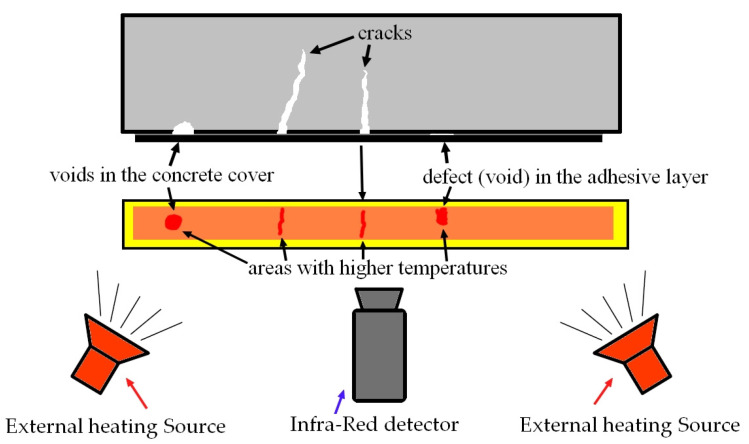
Scheme of using thermography to detect defects in building structures.

Infrared thermography (IRT) is a fairly well-known diagnostic method. It is typically associated with investigating the impact of climatic parameters on a building, but its application is much broader [12,13,14]. The method’s operating principle is quite simple, as it involves measuring the temperature distribution across the recorded surface. Observed differences indicate uneven heat diffusion, and such anomalies in a structural element or material may indicate defects such as voids, cracks, or delamination. The main advantages of this method include ease of use, the ability to conduct real-time inspections, and non-contact measurements. In the case of concrete or reinforced concrete structures, infrared thermography is used to detect cracks [15,16,17]. However, such tests have shown certain limitations, including sensitivity to external conditions such as wind, rain, sunlight, etc. To reduce such interference, active thermography (Figure 1) is recommended, which involves applying an independent heat source, a thermal effect, to the area examined. This measurement involves recording the response of the observed material to thermal effects generated for this purpose (e.g., halogen lamps) using a thermal imaging camera. Due to the small size of the defects, another limitation arises: the small examination area. The resolution of these cameras is not high, so achieving high accuracy requires a reduced distance from the detected surface. The research presented in [16] showed that in the case of concrete structures, the method allows the detection of internal cracks (only at a shallow depth). However, thinner cracks with a width of less than 0.5 mm have been shown to only be observed with an additional stimulus, that is, water.

This method has also been used in the diagnosis of FRP composite materials themselves. An example is the use of long pulse thermography (LPT) to detect both depth and impact defects in GFRP materials [18]. These studies also used image processing techniques to classify composite defects and automatically highlight defective areas. Active thermography can also be used in this type of research, as demonstrated in [19].

Currently, this method is increasingly used to locate defects in concrete elements reinforced with FRP materials [3,20]. Infrared thermography allows in this case to detect invisible cracks, hidden under the glued FRP material as described in [21,22], as well as other types of voids [23]. In this type of research, it should be noted that we are dealing with three materials with significantly different thermal diffusivities: CFRP strip, resin, and concrete. CFRP conducts heat very well, and, for example, in the studies presented in [24], the thermal diffusivity of the tested material along the CFRP fibres was in the range of 240–280.2 × 10^−6^ m^2^/s. This also contributes to the rapid temperature drop in the defect area when the heat source is disconnected. It should also be remembered that this material is anisotropic, meaning that in the second direction, this value is over 100 times lower. Epoxy resin, on the other hand, has the lowest thermal diffusivity, which means that it acts as an insulator; an example range of values obtained in the studies presented in [25] was 0.7–1.1 × 10^−7^ m^2^/s. Concrete has moderate diffusivity and conducts heat better than resin, but still relatively slowly. In [26], the authors, based on their research, present thermal properties of concretes of various strength classes. The obtained thermal diffusivity values ranged from 0.69 to 1.34 × 10^−6^ m^2^/s. The thermal conductivity of both resin and concrete is significantly greater than that of the air filling the defects, so in the area where they occur, we can observe material’s temperature changes—a hot spot occurs, a higher temperature area. This means that as the volume of the void increases, the area and temperature recorded by the camera will also increase.

### 1.2. Ground-Penetration Radar (GPR)—Description of the Method

GPR is a geophysical method that involves emitting an electromagnetic wave into the interior of the medium being examined and then recording the reflected wave. The evaluation of the internal structure of the medium is based on the analysis of the properties of the reflected wave, primarily the amplitude and propagation time. Generally, wave reflection from an object within the medium results in a local increase in amplitude.

Although the first applications of GPR involved assessing the structure of near-surface geological layers [27], in the late 20th century, with the widespread introduction of high-frequency antennas and advanced data analysis methods, GPR was introduced in other areas, including construction. It is particularly often used to inventory the internal structure of concrete structures [28,29,30], as well as to assess the moisture content [31,32] and the degree of corrosion [33,34,35]. The GPR technique can also be used to diagnose structures made entirely of FRP composites [36].

In recent years, GPR has been increasingly used for the non-destructive evaluation of externally reinforced concrete structures with composite elements [3,37]. Most authors claim that high-frequency GPR antennas (1–2 GHz) are effective for detecting subsurface voids in concrete [38,39,40].

Riad et al. [41,42], found that GPR with a 2.6 GHz antenna accurately detected voids and discontinuities at the FRP strip–concrete interface. Furthermore, the authors observed the influence of other bonding parameters (FRP type, number of epoxy layers, epoxy type, surface roughness, etc.) on the reflected wave amplitude. In contrast, the authors of [43] noted that the GPR was useful only in detecting voids, while other defects (for example, delaminations resulting from improper surface preparation before bonding) were not visible.

Ortiz et al. [43] pointed out the practical limitations of using GPR to study CFRP external reinforcements. Carbon fibres, as a conductive material, constitute a barrier to radio waves, making detection of subsurface defects impossible. This limitation does not apply to insulating composites. For example, Li and Liu [44] demonstrated that a 10 GHz microwave system is effective in mapping the structure of concrete reinforced with GFRP mats, including detecting voids and interface delaminations. Similar conclusions were drawn in [45].

However, some authors [38] emphasise that GPR is not a fully reliable tool and should be combined with other NDT methods, such as infrared thermography (IRT).

### 1.3. Wave Velocity Measurement Method (WVMM)—Description of the Method

Nondestructive diagnostic methods are increasingly popular due to the fact that they do not generate structural damage during testing. A group of methods is based on the analysis of acoustic waves at various frequencies and their behaviour within the tested element. Examples include ultrasonic methods [46,47,48]. The second group includes methods using acoustic emission [49], which include, among others, the IADP method [50], based on the recording of acoustic waves generated by active destructive processes occurring automatically, for example, from shrinkage [51,52,53] or under load [54,55] within the element. This method is also used to analyse various materials, including, in addition to concrete [51,52,53] and steel [56,57], composite materials [58] and even soil [59,60]. The diagnostic method used in this study, the WVMM wave velocity measurement method, is also based on the analysis of acoustic elastic waves. In this case, unlike the IADP method, an acoustic signal in the form of an elastic wave is generated externally. This can be used to detect and confirm the location of defects. In this case, the reference Hsu-Nielsen method (Hsu-Nielsen-Source) was used as the wave source, as described in the European [55] and American [61] standards.

The Hsu-Nielsen method involves the generation of an elastic wave as a result of fracture of a graphite stylus with a diameter of 0.5 mm and a hardness of 2H, the protruding part of which should be 3.0 ± 0.5 mm long. During fracture, the stylus generates an impulse with an amplitude of approximately 1.6 N [62]. Furthermore, to achieve the most repeatable fracture positions, a Teflon ring is applied to the tip of the mechanical pencil, which, together with a guide, allows the pencil to be positioned at the same 30 ° angle during the test.

The HSU–Nielsen source has its advantages:

- A broad and flat frequency band ranging from 40 to 400 kHz, similar to the acoustic emission resulting from concrete cracking [63].
- The generated signal is strong, allowing this source to be used for wave measurement at various distances, including measuring the attenuation of longitudinal wave velocity [63,64].
- Simplicity of construction, universality, and ease of use both in the laboratory on samples and in the field on existing structures [63,64].
- Allows for determining the reference wave velocity in concrete [64].
- Allows for checking the sensor connection to the concrete and its correct operation; easy to use [64].

The HSU–Nielsen source also has the following disadvantages:

- Two sensors are required to measure the longitudinal wave velocity, as the source is not synchronized with the measuring equipment, which complicates analysis [63].
- A broken graphite guide may reflect off the sensor or the surface of the element, causing additional light and erroneous results [63].
- The person breaking the guide should maintain a constant pressure, but in practice this is difficult, and each break of the graphite is not always identical [63].
- Material costs increase with the number of tests; pencil lead must be purchased only from a recommended company with the appropriate parameters [63,64].
- Not suitable for tests that require high precision [65].

## 2. Materials

### 2.1. Materials Used to Prepare Specimens and Their Properties

The six identical beams were made, each 0.5 m long and with a square cross section of 0.10 m × 0.10 m. The elements do not have steel reinforcement. All beams were cast at a similar time) in several-day intervals because of the precast form, allowing for the production of 4 elements/beams at one time. The concrete samples strength test was carried out in parallel to the beam tests. Concrete average cylinder compressive strength f_c_ = 49.4 MPa hence the designated concrete class is C45/55. The concrete recipe is given in Table 2.

As strengthening, a CFRP strip manufactured by Sika, designated Sika^®^ CarboDur^®^ S 512 and Sikadur-330 resin were used (basic material properties are given in Table 3 and Table 4).

### 2.2. Preparation of the Specimens

**Figure 2:**
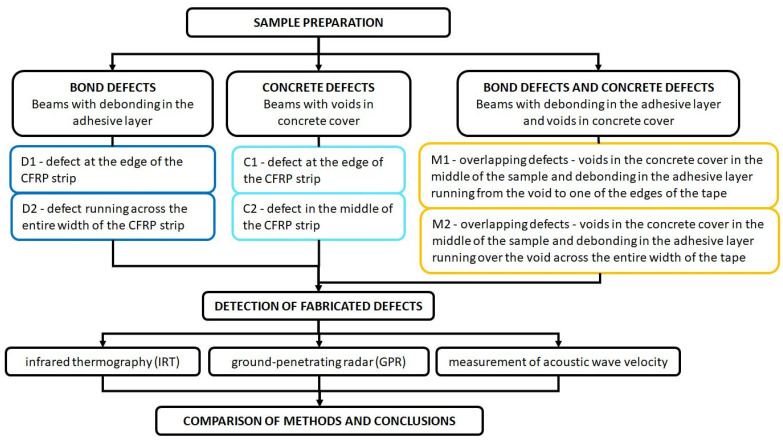
Flow chart of the research methodology.

The beams were divided into four groups, three of which contained defects (Figure 2):

- N1 and N2—beams without defects;
- D1 and D2—beams with a bond defect in the adhesive layer;
- C1 and C2—beams with a concrete defect in the form of drilled voids in the concrete cover;
- M1 and M2—beams with overlapping defects of both types.

The preparation of the strengthened specimens was the same for each beam. It required several steps:

1. Grinding and cleaning the concrete surface of the samples;
2. Preparation of defects—voids in the concrete cover in samples marked with symbols by drilling holes in accordance with the drawing using a hammer drill with a drill bit of 10 mm diameter;
3. Cleaning the surface of the CFRP strip;
4. Applying a thin layer of resin to the prepared surface and spreading it with a putty knife to fill all pores, avoiding defects. Removal of excess resin with a putty knife;
5. Attaching a plastic strip to the place of simulated defects (15 mm wide);
6. Applying the resin to the strip using a previously prepared trough-type tool with dimensions consistent with the manufacturer’s requirements;
7. Adherence of the CFRP strip to the prepared concrete surface and pressing it to remove excess resin;
8. Sliding the plastic strip out from under the CFRP strip, creating a void in the resin layer between the strip and the concrete surface;
9. Removing the excess resin using a putty knife.

**Figure 3:**
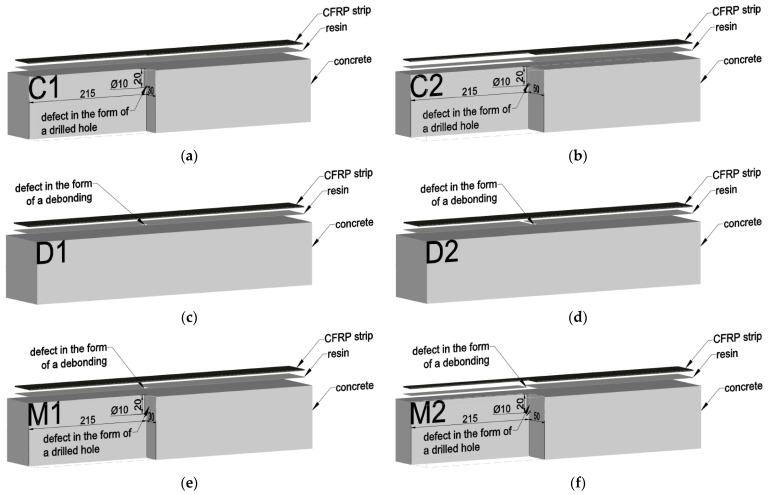
Location of defects in beams (**a**,**b**) defects in the form of a hole under the composite–concrete defects, (**c**,**d**) defect in the form of debonding–bond defect in the adhesive layer, (**e**,**f**), samples with both types of damage—units are given in [mm].

**Figure 4:**
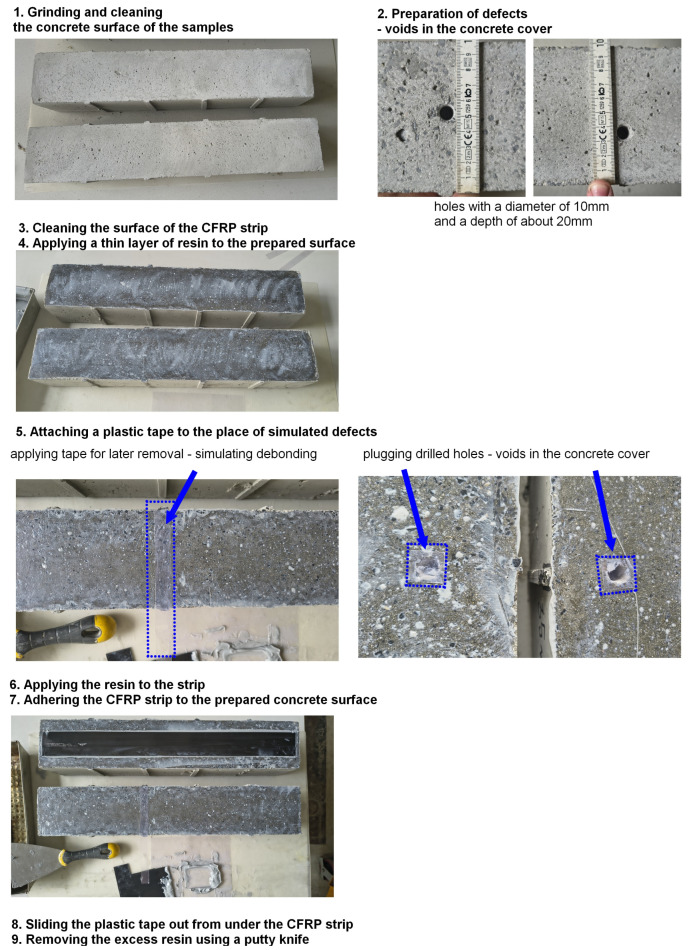
Preparation of the strengthened specimens.

**Figure 5:**
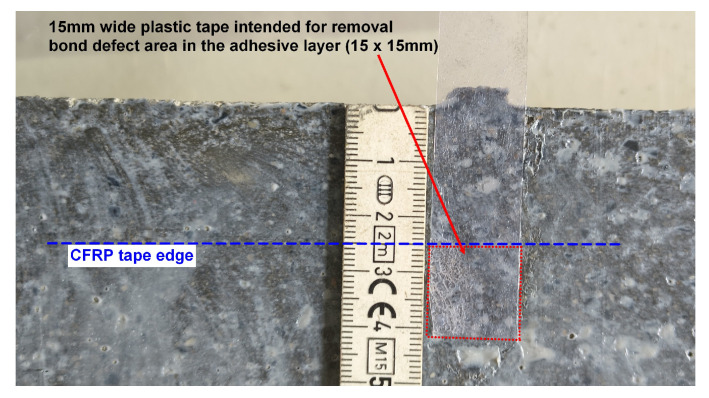
Dimensions of the bond defect area in the adhesive layer for sample D1.

The type and location of defects in the individual beams are shown in Figure 3. Photos of the subsequent stages of the concrete beam are shown in Figure 4 and Figure 5.

Proper surface preparation of the structural element before bonding FRP materials is a critical step in ensuring the effectiveness of the reinforcement. When executed correctly, it can significantly delay debonding and improve the ultimate strength of the system. Key preparatory actions include removing any loose concrete, levelling the surface to eliminate irregularities, and thoroughly cleaning both the concrete substrate and the FRP material to remove contaminants. It is equally important that the epoxy resin remain clean and uncontaminated. It is also possible to apply additional treatments to the prepared surface, which is described in more detail in [68]. These treatments include transverse, diagonal, and longitudinal grooving. They aim to increase the contact surface area and further help to postpone the onset of debonding.

In the case of strengthening real structures, concrete surface preparation should be preceded by verification of the concrete substrate’s soundness. Depending on the requirements, the minimum concrete tensile strength should be greater than 1.5 N/mm^2^ (PN-EN 1542 [69]) or 1.4 N/mm^2^ (ACI 440.3 L.1 [70]). Rebar corrosion should be stopped to avoid damage to the concrete cover due to extensive rust. The depth of carbonation and chloride content (concentrations greater than 0.3% by weight of cement are assumed dangerous) should also be verified. According to the requirements described in [9], horizontal cracks, cracks wider than 0.2 mm, and holes in the concrete cover should be filled or sealed with injection. This aims to reduce the risk of reinforcing steel corrosion, solve leakage problems, and avoid weak bond strength.

**Figure 6:**
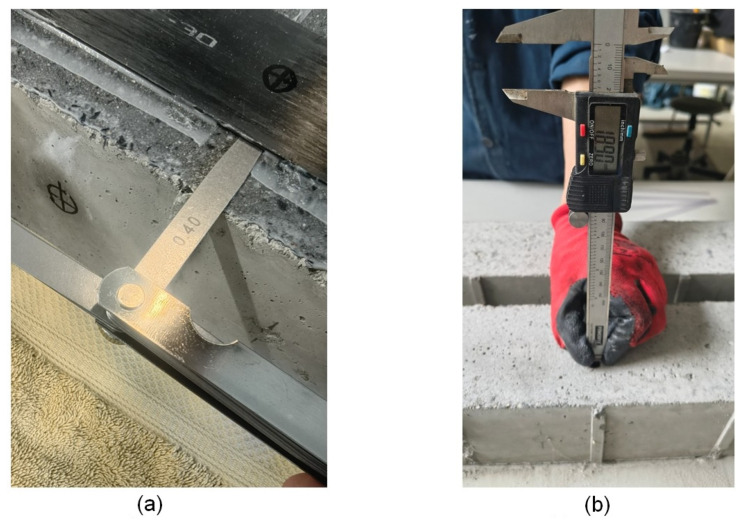
Example measurement of defect size: (**a**) thickness of the defect in the adhesive layer; (**b**) concrete defect in the form of drilled voids in the concrete cover.

Before testing, the thickness of bond defects/voids in the adhesive layer was measured for samples D1, D2, M1, and M2 using a feeler gauge. The depth of concrete defect/drilled voids in the concrete cover for samples C1, C2, M1 and M2 was measured using a digital caliper. The results are summarised in a Table 5, and a sample measurement is shown in Figure 6.

## 3. Methods and Results

### 3.1. Infrared Thermography (IRT)

To detect defects, active thermography was used; i.e., an external heat source was used to heat the CFRP strip in the area captured by the thermal imaging camera.

**Figure 7:**
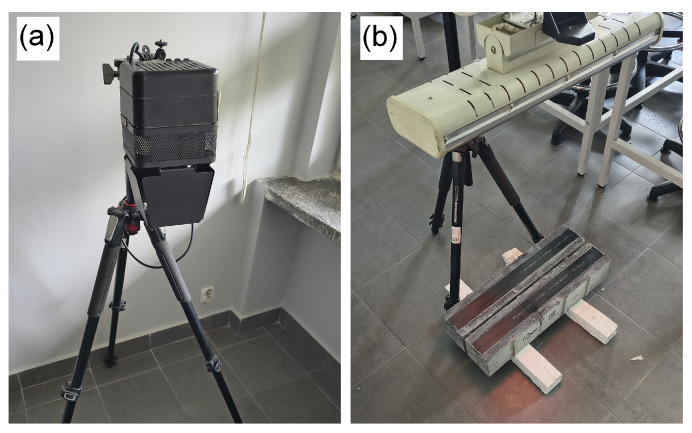
Heat sources used in the test: (**a**) halogen lamp; (**b**) radiant heater.

**Figure 8:**
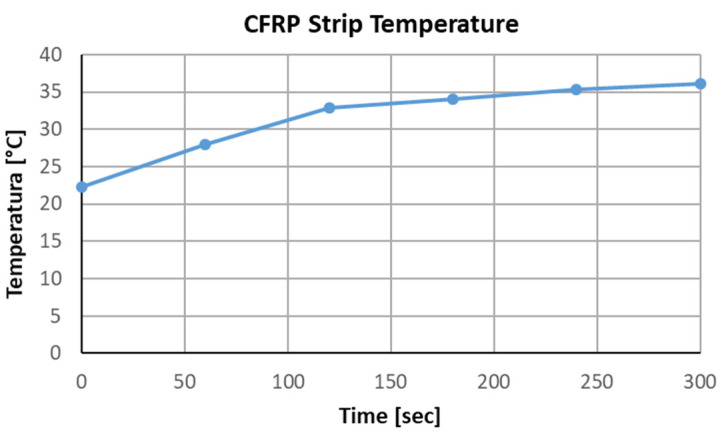
Averaged results for the temperature increase in the central part of the CFRP strip during heating.

The surface of the specimens was heated using two types of radiation sources: a halogen lamp (KFB 93325, 2 × 1000 W, Kaiser Fototechnik GmbH & Co. KG, Buchen, Germany) and an infrared radiant heater (Einhell QHT 1500, 1500 W, Einhell Germany AG, Landau/Isar, Germany) (Figure 7). Both lamps were placed at a distance of about 1 m from the surfaces of the specimens. The heating duration was approx. 5 min. It allowed the CFRP strip to reach a temperature of approximately 37–38 °C. A radiant heater was placed above the samples perpendicular to the surface with the CFRP strip applied, so that the strip, not the concrete sample, was heated. The short heating time was intended to limit the amount of heat supplied to increase the concrete temperature. The scale effects associated with the small size of the concrete sample were negligible, as smaller samples heat up faster but have a higher surface area relative to their volume, which means that heat dissipates more efficiently and the temperatures obtained for the concrete should be similar. In the case of CFRP strip, which has the highest thermal conductivity, the scale effect may be much more significant, but this requires confirmation on full-size samples. Before testing, the CFRP strip surface was cleaned using isopropyl alcohol. This is important to remember, as any contamination, such as dust or grease, can cause uneven surface heating. The temperature increase in the central part of the CFRP strip during heating was recorded by an additional thermal camera (UNI-T Uti260B, Uni-Trend Technology Co., Ltd. Dongguan, China) every minute. The average results are presented in Figure 8.

The ambient temperature during the test was 22 ± 1 °C and the emissivity of the concrete surface was set to 0.95 on the infrared camera (Testo 890, Testo SE & Co. KGaA, Black Forest, Germany). Air humidity was 31.5%, measured using a Benetech GM1365 humidity and Temperature Recorder.

Preliminary tests showed that the halogen lamp heated the surface unevenly, making defect localization difficult. In contrast, the infrared radiant heater provided a more uniform and controllable thermal excitation, and was therefore used in subsequent measurements. In addition to power, the heating efficiency depends on the spectral characteristics of the source, the distance and angle of incidence, and the surface properties of the specimen. Although the halogen lamp provides higher luminous flux, its emission in the near-infrared range results in less effective and less uniform heating compared to the infrared radiant heater.

**Figure 9:**
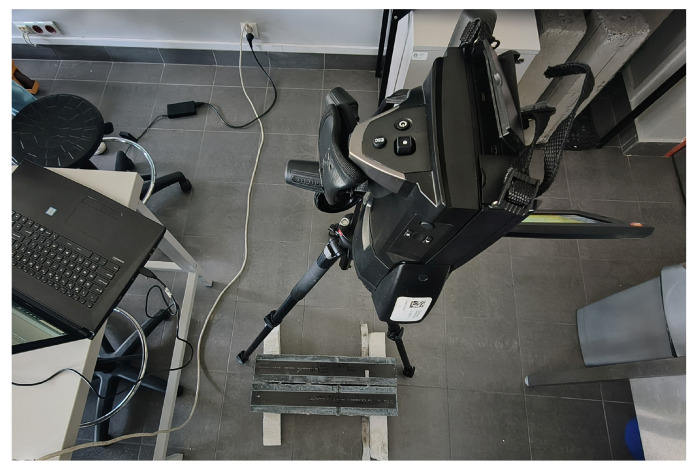
A thermal imaging camera placed above the test samples.

A Testo 890 thermal imaging camera with an infrared resolution of 640 × 480 pixels (Figure 6) and other technical parameters specified in Table 6 were used for the measurements. It was placed on a tripod approximately 120 cm above the samples (Figure 9).

**Figure 10:**
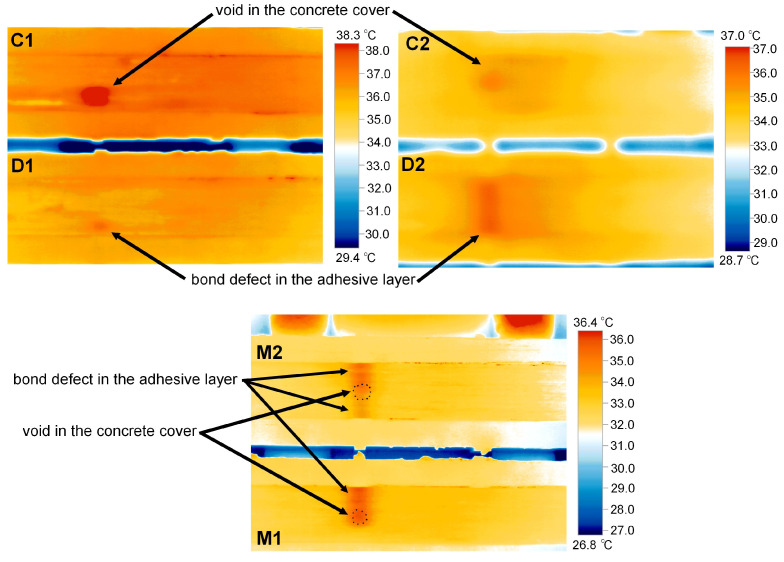
Infrared data obtained using the IRSoft software for the tested sample.

**Figure 11:**
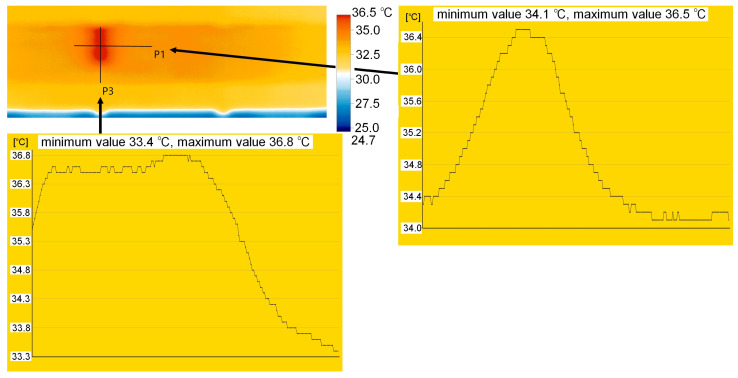
Temperature distribution along selected sections for sample M1.

**Figure 12:**
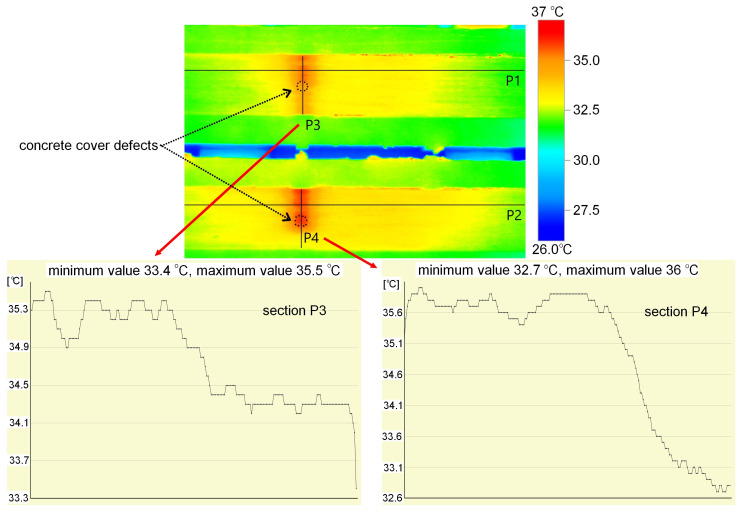
Results of temperature distribution measurements on the surface of samples M1 and M2—repeated testing.

The measurement, that is, the image was recorded immediately after the heat source was removed. The defects were visible in the image for approximately 10 s, after which the temperature difference disappeared very rapidly. The results obtained, along with the defect indications, are presented in Figure 10. Images obtained from a thermal imaging camera allow for the location of defects, but only for a short time after the heat source is removed. All defects were detected, regardless of type and volume. The temperature within the defects is approximately 2 °C higher. This is clearly visible in the detailed analysis presented for sample M1, where cross sections were made as shown in Figure 11. The thickness of the defect in the adhesive layer in this case is 0.65 mm. The temperature readings are presented in the graphs shown in Figure 8. This analysis also allows us to observe a small temperature difference between overlapping defects, approximately 0.2–0.3 °C. A similar effect is visible in the case of sample M2, where varying defect thicknesses in the adhesive layer in the adhesive layer (from 0.4 to 0.65 mm) are observable. Higher temperatures—about 1 °C (from 34.3 °C to 35.3 °C, section P3 in Figure 12)—can be observed on the side with the larger defect thickness. However, these are very small differences that may be difficult to observe in a real structure due to contamination or environmental influences. The defect in the concrete cover is not visible in this sample. Re-examination of samples M1 and M2 carried out to verify the repeatability of the obtained results and a broader analysis of the possibility of locating overlapping defects gave similar results (Figure 12). Again, in the case of sample M1, slightly higher temperature values were obtained in the hole area of the drilled in the concrete cover. The maximum recorded temperatures within the defects for both samples were similar: 36 °C (sample M1) and 35.5 °C (sample M2). Both occurred in locations where the thickness of the defect in the adhesive layer was similar, at 0.65 mm. In sample C2, the temperature in the bond defect area was higher than in the concrete defect area of sample D2. On sample D1 (Figure 10), a lighter area can be seen to the left of the defect; this is where the strip was contaminated with resin during sample preparation.

The main factor influencing the increase in visible temperature in the area of defects is a significant change in the thermal conductivity of the material properties under the CFRP strip, meaning that instead of resin and concrete, air is filling the defects. The change in depth and, therefore, the volume of air in the defect, contributes to very small deviations in the recorded temperature, which may well result from other factors, such as surface contamination or concrete moisture.

All infrared data were analysed using the IRSoft v5.2 software (Testo SE & Co. KGaA, Germany). The software allowed the extraction of temperature profiles, contrast enhancement, and emissivity correction. The thermograms shown in Figure 10, Figure 11 and Figure 12 were exported directly from IRSoft.

### 3.2. Ground-Penetrating Radar (GPR)

Regardless of the tests described above, an attempt was made to detect defects in the strip-sample surface connection using ground-penetrating radar (GPR). IDS Aladdin GPR system with a 2 GHz antenna was used. The choice of this type of antenna was justified by its wide practical application and compromise between measurement accuracy (antenna resolution) and depth range. Commercially available antennas with higher frequencies (up to 6 GHz) are characterised by higher resolution and accuracy, but their short range (on the order of several centimetres) makes their use ineffective in engineering applications.

**Figure 13:**
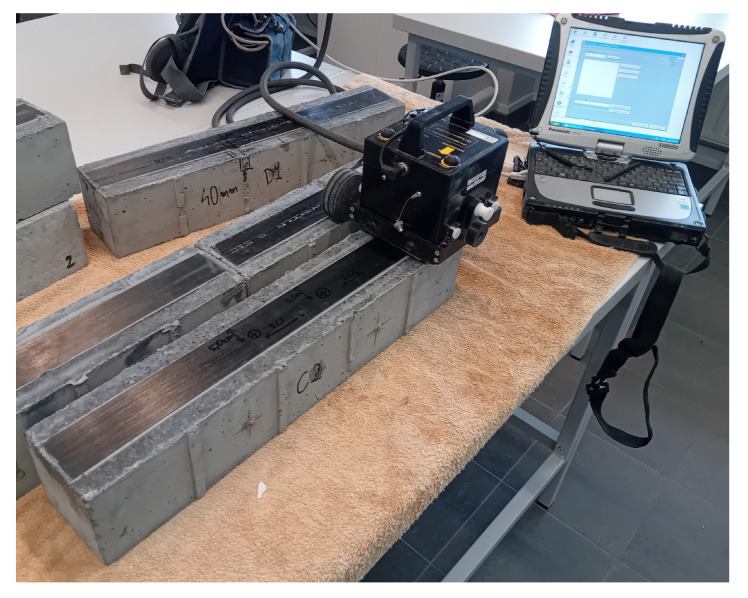
Scanning the sample using the IDS Aladdin georadar and a 2 GHz centre frequency antenna.

Each experimental study involved passing the antenna along the test sample, directly over the defect. The studies were carried out in two variants: scanning the surface of the composite strip (Figure 13) and scanning the opposite (unreinforced) surface.

To ensure the good quality and repeatability of the results, the scanning was repeated five times in two directions.

**Figure 14:**
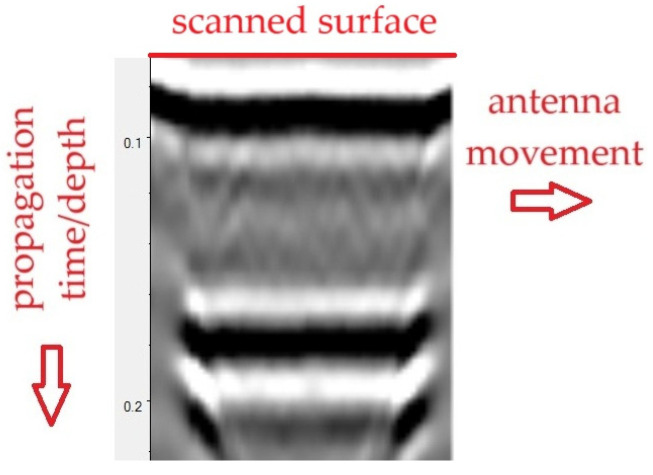
Interpretation of GPR results.

The results in the form of radargrams (Figure 14) were analysed. The radargram can be interpreted as a “vertical cross section” of the tested object, placed along the antenna’s path. The upper horizontal axis of the radargram represents the antenna’s path across the tested surface. The vertical axis, in turn, represents the time of bidirectional wave propagation (from the transmitting antenna to the medium and back). Knowing the wave propagation velocity in the tested medium, one can convert the time scale to a depth scale, allowing one to determine the depth of the target object. In the case of concrete, the wave propagation velocity is approximately 10–13 cm/ns.

The greyscale in Figure 14 presents the amplitude of the wave reflected from individual areas of the medium. Negative amplitudes are marked in black, whereas positive amplitudes are marked in light colours. Discontinuities and objects within the medium are visible as local peaks in wave amplitude.

**Figure 15:**
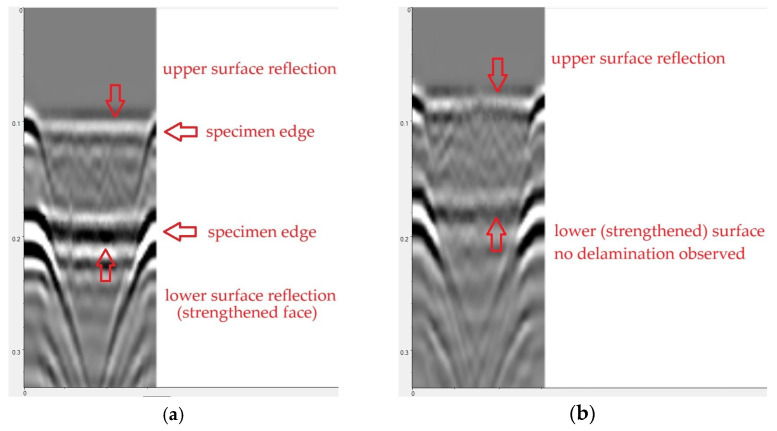
Example radargrams recorded during sample scanning: (**a**) sample without defects (N2), (**b**) sample with CFRP strip debonding (D1).

Examples of processed radargrams are presented in Figure 15. In each case, horizontal bands are visible, representing wave reflection from the top and bottom surfaces of the sample. Inverted hyperbolas can be observed at the beginning and end of each scan, indicating wave reflection from the edges of the sample.

Figure 15a illustrates an example radargram recorded on a defect-free sample (N2), In this case the antenna was moved across the unreinforced surface of the sample and the reinforced surface was located underneath. As mentioned above, the horizontal bands in Figure 15a indicate wave reflection from the sample surfaces. No signals were observed between the bands (in the sample volume). Therefore, no defects were detected, either in the concrete volume or in the reinforced area.

Figure 15b presents an example scan result for a sample with an adhesive layer defect (debonding, sample D1). As before, the scan was conducted on the unreinforced surface, so the surface with the strip and adhesive bond is visible in the lower part of the radargram. However, none of the measurements recorded a signal that could indicate the presence of a defect in the adhesive layer. This is due to the small size of the defect (approximately 2 mm thick) relative to the antenna resolution.

The vertical resolution of the antenna (the ability to distinguish objects located at different depths) can be estimated as 0.25 of the wavelength λ [71]. The value of λ is in turn, defined as the quotient of the wave speed to the centre frequency. Assuming a typical wave speed for concrete *v* = 10 cm/ns = 10^8^ m/s and the central frequency of the antenna used *f* = 2 GHz = 2·10^9^ Hz, one can obtain:

(1) $$\lambda =\frac{v}{f}=\frac{10^{8}}{2\cdot 10^{9}}=0.05m=5cm,$$

The vertical resolution R can be calculated as follows [72]:

(2) $$R=\frac{\lambda }{4}=\frac{5}{4}=1.25cm,$$

As can be seen, the resolution of the antenna used is much greater than the size of the defect in the adhesive layer. In general, it is assumed that GPR allows for the detection of objects measuring at least the antenna resolution determined by Equation (2). As discussed in [72], the detection of smaller objects is possible in principle but requires high-quality input data as well as complex processing techniques. In practical terms, it can therefore be assumed that the limiting size of a void that can be detected using a 2 GHz antenna is approximately 1–1.5 cm.

Furthermore, the dielectric properties of concrete are crucial for estimating resolution. These properties change, for example, depending on the humidity or the presence of chlorides. These factors directly influence the dielectric constant *ε*_r_, which is a measure of its ability to store energy in an electric field. A change in *ε*_r_ (resulting, for example, from concrete moisture) directly translates into a change in wave velocity, according to the formula:

(3) $$v=\sqrt{\frac{c^{2}}{\varepsilon_{r}}},$$

where *v*—mean wave velocity in the tested medium, *c*—speed of light in vacuum, *ε*_r_—dielectric constant of concrete.

Since increasing the moisture content of concrete leads to an increase in the dielectric constant (*ε*_r_ for concrete is between 9 and 12, while for water it is 81), according to Equation (3), the wave velocity decreases, which in turn results in a reduction in wavelength (Equation (1)) and improved resolution (Equation (2)). In practice, this means that smaller objects can be detected but at the cost of increased wave attenuation and reduced range.

Generally, however, objects smaller than the theoretically determined antenna resolution are not visible as a strong, single echo, but rather as a small wave disturbance that can easily be confused with background noise.

**Figure 16:**
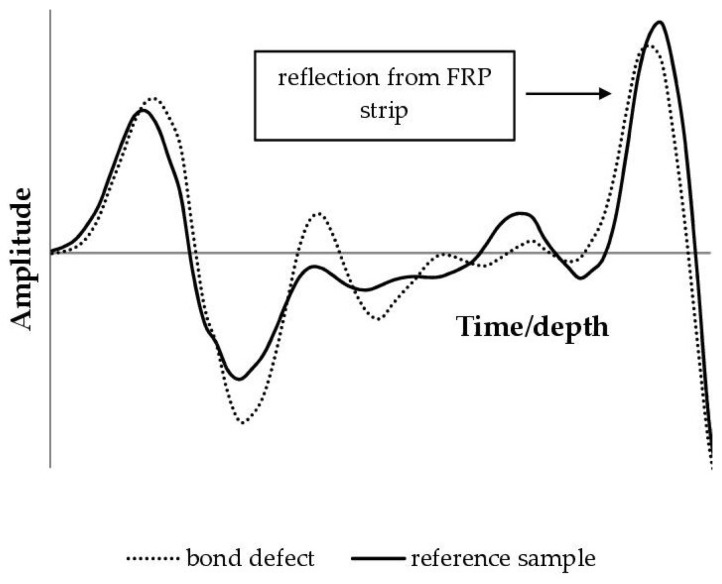
Influence of the bond defect on the amplitude of wave reflected from the CFRP strip.

Taking the above into consideration, delamination can affect the characteristics of the reflected wave (namely, reducing the wave amplitude). This problem has been described in some detail for the case of reinforced concrete slabs in bridges [73]. In this paper, on this assumption, an attempt was made to evaluate the effect of a bond defect on the amplitude of the wave reflected from the CFRP strip. Figure 16 presents the results obtained for the case where scanning was performed on an unreinforced surface and the wave propagated through the thickness of the sample. The wave reflection from the strip is visible on the right side of the graph. In debonding, the peak amplitude was slightly lower (3.26 V) than in defect-free areas (3.42 V).

Although visible and repeatable in all tested samples, this effect is not strong enough to be of practical use. Therefore, the 2 GHz GPR antenna is not a useful tool for assessing adhesive layer defects in joints.

**Figure 17:**
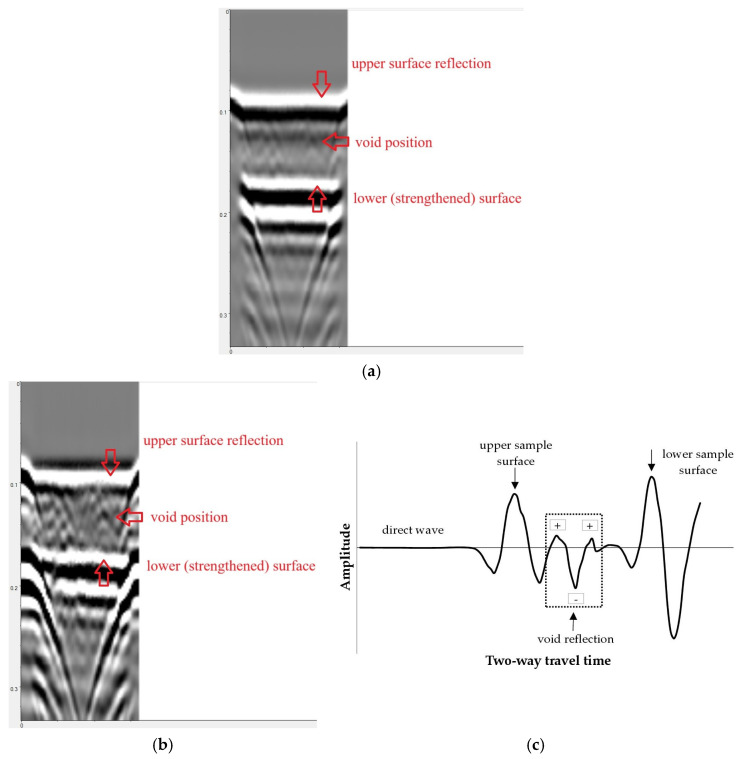
Radargrams recorded on sample with a void under the strip (C1): (**a**) raw data, (**b**) radargram form (**a**) after background removal and contrast enhancement, (**c**) A-scan through void section.

Figure 17 presents the results of scanning C1 sample (with a void in the concrete under the strip). Scanning was performed on the unreinforced surface.

Figure 17a illustrates a raw radargram (without filters or processing). As before, wave reflections from the sample surface and edges are clearly visible. In addition to these signals, an echo in the form of an inverted hyperbola is visible in the right part of the radargram, originating from a void in concrete. However, due to environmental noise and multiple reflections in the radargram, this signal is poorly visible and can easily be missed during interpretation.

Several radargram processing procedures were used to highlight this echo, namely: background removal, bandpass filter, Hilbert envelope, and linear gain. The void was the most clearly visible in radargrams subjected to background removal, possibly combined with linear gain or contrast enhancement. Figure 17b illustrates an example radargram after applying background removal and increasing contrast. The hyperbola representing the wave reflection from the void is clearly discernible.

This conclusion is confirmed by analysis of the polarity of the reflected wave. As can be seen in Figure 17b, the reflection from the void actually includes 3 hyperbolas (looking from top to bottom, the hyperbola is white, the one below is black and then white again). This means that the signal includes 3 amplitudes in the order +/−/+. The negative (middle) amplitude has the highest value. In the case of the system used, this means that the wave was reflected, passing from a medium with a higher dielectric constant *ε*_r_ to a medium with a lower constant. Since the value of *ε*_r_ for concrete is 9–12 and for air it is 1, the result obtained indicates that the wave was most likely reflected from a void filled with air.

The behaviour of the wave after reflection can also be analysed in terms of the reflection coefficient *Γ*. It is defined as the ratio of the amplitude of the wave reflected from the object *A*_ref_ to the amplitude of the incident wave, *A*_inc_. While *A*_ref_ is directly read from the radargram, accurate determination of *A*_inc_ is not possible in practice. A certain approximation of *A*_inc_ can be the amplitude of the wave reflected from a metal plate, but such a measurement does not take into account a number of phenomena, such as wave attenuation and scattering in concrete, wave reflection from the sample surface, environmental noise, and others.

Therefore, alternatively, assuming the direction of wave incidence normal to the surface of the object, the reflection coefficientwas determined from the formula:

(4) $$\Gamma =\frac{\sqrt{\varepsilon_{object}}-\sqrt{\varepsilon_{concrete}}}{\sqrt{\varepsilon_{object}}+\sqrt{\varepsilon_{concrete}}},$$

where *Γ*—wave reflection coefficient, *ε*_object_—dielectric constant of the object (void filled with air was assumed, therefore *ε*_object_ = 1), *ε*_concrete_—dielectric constant of concrete.

The value of *ε*_concrete_ was determined by transforming Equation (3) into the form:

(5) $$\varepsilon_{concrete}=\frac{c^{2}}{v^{2}},$$

The wave velocity *v* was calculated as the quotient of the doubled distance from the sample surface to the void (the wave path from antenna to the void and back) divided by the time of bidirectional wave propagation read from the radargram. The average wave velocity between the sample surface and the void was $9.21\;\frac{cm}{ns}$, and the value of the *ε*_concrete_ from Equation (5) was 10.61. Substituting into Equation (4) *ε*_object_ = 1 and *ε*_concrete_ = 10.61, the reflection coefficient was obtained:

(6) $$\Gamma =\frac{\sqrt{1}\;-\;\sqrt{10.61}}{\sqrt{1}\;+\;\sqrt{10.61}}=-0.53,$$

The (−) sign in this case indicates a change in the polarity of the reflected signal.

Since the depth of the void was 3 cm and the estimated resolution of the apparatus was 1.25 cm (Equation (2)), detecting the defect with the 2 GHz antenna was not problematic.

However, due to its small diameter (1 cm), the void should be regarded as a point defect. In this situation, the horizontal resolution of the GPR apparatus becomes particularly important, as it indicates the ability to distinguish objects located next to each other horizontally. The horizontal resolution *R*_h_ depends on the depth of the defect and is determined from the following relationship:

(7) $$R_{h}=\sqrt{\frac{\lambda \cdot z}{2}},$$

where *R*_h_—horizontal antenna resolution, λ—wavelength according to Equation (1), z—depth of defect location.

In the analysed case, assuming *λ* = 5 cm (see Equation (1)), and *z* = 7 cm (distance from the surface of the scanned sample to the void), *R*_h_ can be calculated:

(8) $$R_{h}=\sqrt{\frac{5\cdot 7}{2}}=4.18\;cm,$$

As can be seen, the diameter of the void is significantly smaller than the system’s horizontal resolution. However, unlike vertical resolution, this does not mean that the defect cannot be detected. In this case, the void is visible as a point defect, and its small dimensions compared to the *R*_h_ mean that its shape and extent cannot be determined from the radar image.

Summarising the results of the scan on the unreinforced surface (Figure 15, Figure 16 and Figure 17), it can be concluded that the GPR system was found to be an effective tool to detect voids in concrete located under the composite reinforcement, but it is impossible to diagnose defects and adhesive joint defects using this system. The test results for samples containing both types of defects (void and adhesive joint defect, samples M1 and M2) confirm these conclusions and will not be discussed in detail here.

**Figure 18:**
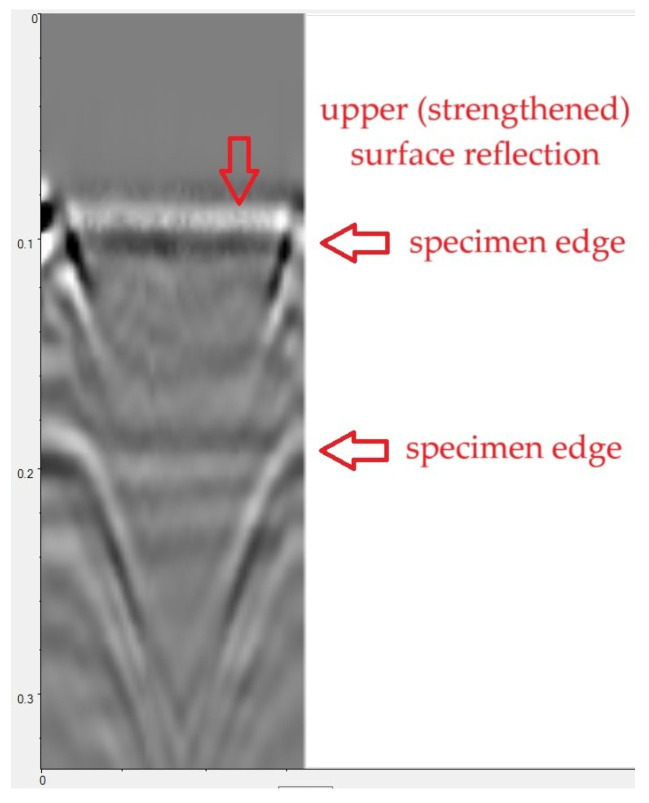
Radargram recorded on sample with a void (C1), scanned on the surface of the composite strip.

Scanning the samples on the composite-reinforced surface did not reveal the internal structure of the concrete sample or any defects in the composite reinforcement. This is due to the fact that the CFRP fibre used in the reinforcing strips is an electrical conductor and therefore reflects electromagnetic waves. As a result, depending on the fiber content antenna frequency and the resin used the dielectric constant of CFRP ranges from 3 to 30 [74] or more. Therefore, the penetration of the wave into the sample is very difficult or even impossible. An example of the results of scanning sample C1 (with a void) from the composite reinforcement side is shown in Figure 18. As can be seen, the reflected signals are weaker than before, and unlike scanning the opposite surface (Figure 17), it was impossible to detect the void.

### 3.3. Wave Velocity Measurement Method (WVMM)

**Figure 19:**
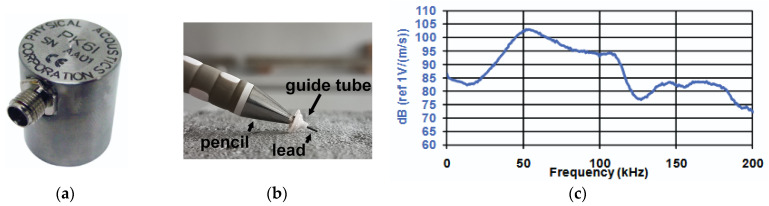
(**a**) PK6I resonant sensor: (**b**) example of a pencil with a guide prepared to generate a wave using the Hsu-Nielsen method (**c**) graph of the sensor sensitivity as a function of frequency [77].

The study used the Pocket AE unit [75], which allows the connection of two sensors and an external input parameter that records the load or stress for correlation with the measurements of acoustic emission. The kit is portable and allows for conducting research both in the laboratory and in the field for up to six hours. The device has an 18-bit analog-to-digital converter, which allows for sampling rates of up to 10 MSPS (Mega Samples Per Second). An interesting feature of the device is the ability to excite the crystal in the acoustic emission sensor using repetitive pulse sequences (Auto Sensor Test). During the study, acoustic wave parameters such as counts, amplitude, energy, rise time, and waveform are recorded and can be analyzed in real-time as graphs. Two PK6I [68] resonant sensors with a built-in 26 dB preamplifier, with operating frequency range 35–65 kHz and peak sensitivity 106 dB (Figure 19a,c) were used in the study, which can both generate and receive acoustic emission waves.

The study (Figure 19b) aimed to measure the elastic wave velocity [76] in concrete and composite materials based on the path between the sensors and the difference in the wave’s arrival time at the sensors. The study was preceded by preliminary work consisting of cleaning and grinding the surfaces to which the sensors were attached. Additionally, a silicone layer was applied to the surface to ensure adequate contact between the sensors and the elastic wave registration surface.

**Figure 20:**
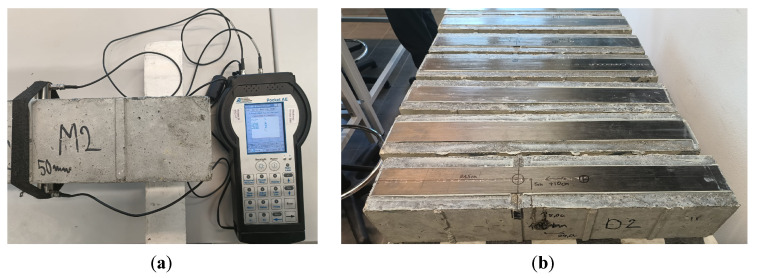
Specimen under the test: (**a**) apparatus during the test; (**b**) specimens prepared to test.

**Figure 21:**
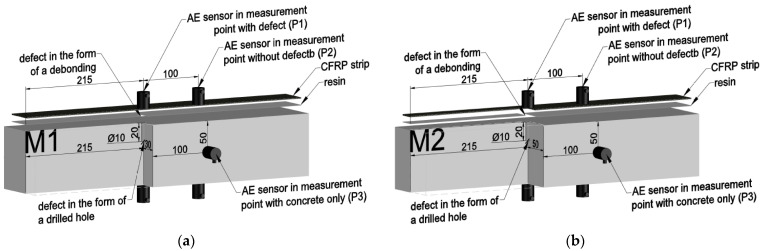
AE sensor arrangement diagram for the M1 and M2 samples; (**a**) M1 sample with a defect in the form of a drilled hole and a detachment under a part of the composite; (**b**) sample with a defect in the form of a drilled hole and a detachment along the entire width of the composite—units are given in [mm].

The tests were carried out on six samples marked with symbols C1 and C2, D1 and D2, and M1 and M2 (Figure 20b), characterized in Chapter 2.2 of this study. Elastic wave velocity measurements were performed for each sample using PK6I sensors located on opposite sample walls, as shown in (Figure 20a), at three measurement points designated on the element surface, enabling the estimation of elastic wave velocity in the assumed cross-sections (Figure 21):

- P1 indicates the measurement point located above the damage;
- P2 indicates the measurement point 10 cm from the damage;
- P3 indicates the measurement point located on the wall perpendicular to the plane with the composite adhered.

The measurement points indicated above (Figure 21) were located to, among other things, obtain the velocity of an elastic wave passing through an intact medium (composed of composite, resin, and concrete), i.e., the so-called baseline elastic wave velocity. They were also used to determine the velocity for a medium composed of the same layers, assuming that various defects were present at the measurement location. Therefore, as already mentioned, the first measurement point (P1) was positioned at the defect location so that the elastic wave passed through the prepared defects. The second point (P2) was approximately 10 cm from P1 so that the elastic wave did not encounter any defects along its path and, as a result, passed through the baseline composite–resin–concrete system, allowing for the baseline elastic wave velocity value to be obtained. The third point (P3) was set so that the sensors were directly adjacent to the concrete surface, hence they were placed on walls perpendicular to the surface on which the composite was attached, as a result of which a comparative value of the elastic wave velocity passing only through the concrete was obtained.

It should also be noted that, regardless of the measurement point, at least one of the sensors was always placed on the concrete, while the other was located on the opposite side. As a result, in the case of points P1 and P2, it was placed directly on the composite surface, and in point P3, it was placed on the concrete (Figure 21).

**Figure 22:**
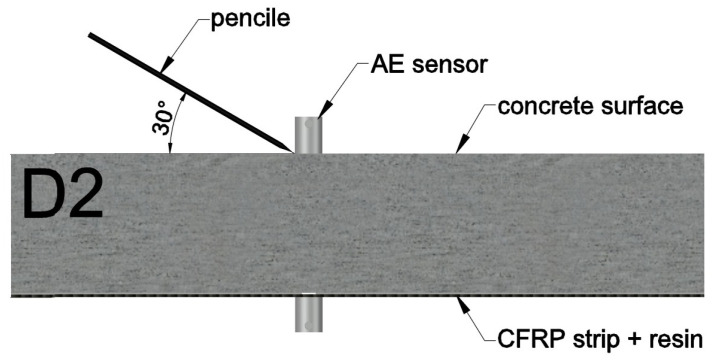
Schematic diagram of elastic wave excitation using sample D2 as an example.

Furthermore, at each measurement point (P1–P3), the elastic wave excitation always took place in the immediate vicinity of sensor no. 1 by breaking a graphite stylus 10 times at an angle of 30°, as illustrated schematically in Figure 22. The average values of the elastic wave speed recorded at the measurement points was shown in Table 7.

**Figure 23:**
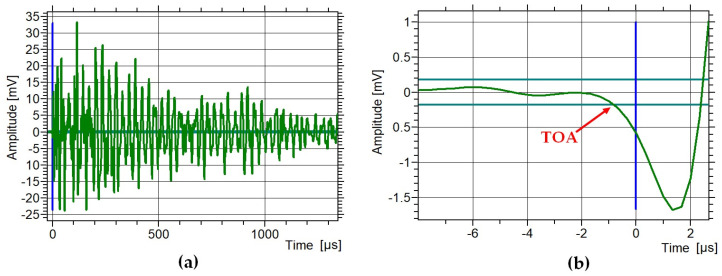
Waveform recorded in the M2 sample without defects: (**a**) full scale, (**b**) beginning of the wave with the red marked time of arrival (TOA).

**Figure 24:**
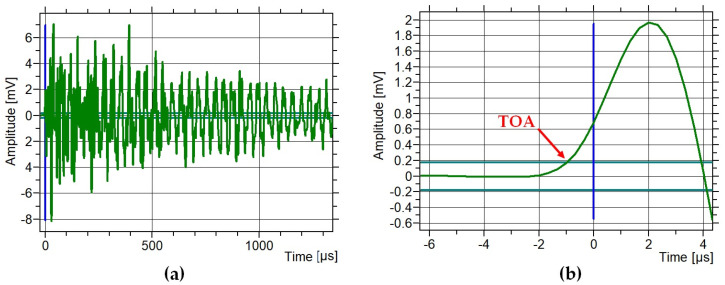
Waveform recorded in the M2 sample with defects: (**a**) full scale, (**b**) beginning of the wave with the red marked time of arrival (TOA).

The time between the two sensors was determined using the standard threshold method. In all experiments, the threshold was set to 45 dB, which corresponds to 0.18 mV after conversion. Figure 23 and Figure 24 present example waveforms recorded on the side opposite the pencil-lead break (Hsu–Nielsen test). The time of arrival (TOA) was defined as the moment of the first threshold crossing of the wave, as shown in Figure 23b and Figure 24b. Figure 24a shows a waveform recorded in the M2 sample without defects. The peak amplitude rises quickly to about 35 mV and then rapidly decays to about 5 mV. Figure 24b shows the first wave peak, which drops to −1.5 mV. Figure 24a shows a waveform recorded in the M2 sample with defects. The peak amplitude rises quickly to about −8 mV and then slowly decays to about 2 mV. Figure 24b shows the first wave peak, which increases to 2 mV. Based on Figure 23 and Figure 24, clear differences can be observed between the waveforms recorded in defect-free and defective samples. Therefore, waveform analysis can serve as an additional indicator of structural changes in the sample.

The elastic wave speeds obtained in this way allowed for estimating the average values of the elastic wave speed (V_w_) together with the basic statistical parameters in the form of standard deviation (s) coefficient of variation (CV_w_) and standard error of the mean (SE) (Table 7). It should be mentioned here that the obtained results were very good repeatability, because in most cases the determined coefficient of variation was within the range of 1–4%, reaching a maximum value of no more than 9%. Also a low standard error of the mean (SE) indicates that the mean obtained from the analyzed sample corresponds with a high probability to the actual population mean. The mean wave velocity V_w_ values in concrete (P3) were consistent between 3650 and 3750 m/s, with the exception of sample M2, which the authors attribute to improved concrete compaction. This confirms that the observed decreases in mean wave velocity in P1 are primarily due to a defect in the CFRP/adhesive/concrete propagation path, rather than to the overall quality of the concrete. It was also noted that the larger and more extensive the defect, the greater the decrease in V_w_ velocity over the defect (P1) relative to the defect-free reference (P2):

- D1 (debonding at the edge of the CFRP strip): ΔV_w_ = 2% → barely measurable effect.
- C1, C2 (voids at the edge and in the middle of the CFRP strip): ΔVw = 10–14% → moderate decrease.
- M1, M2 (overlapping defects): ΔV_w_ = 18–19% → significant speed drop.
- D2 (debonding running across the entire width of the CFRP strip): ΔV_w_ = 21% → greatest speed drop.

**Figure 25:**
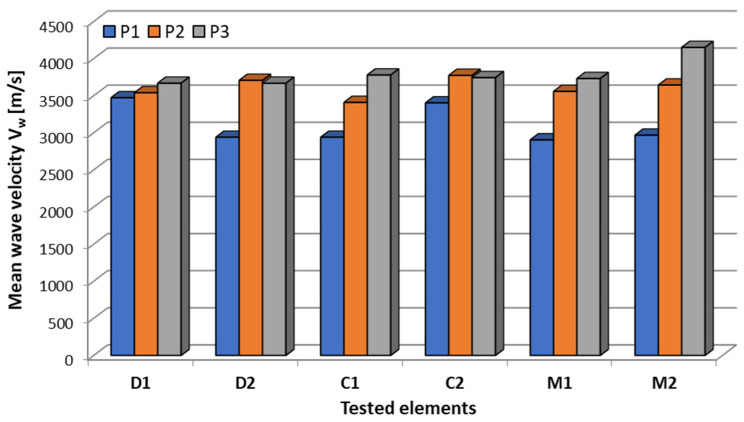
Comparison chart for the obtained average values of elastic wave velocity V_w_ for individual samples at each of the three measurement points P1 (measurement through the composite at the effect location), P2 (measurement through the composite at the defect-free location), P3 (measurement only through the concrete).

**Figure 26:**
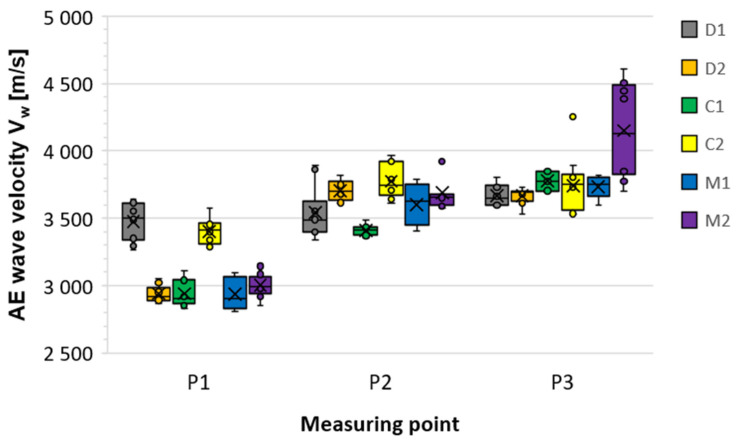
Comparison chart for the obtained average values and statistical parameters of elastic wave velocity V_w_ for individual samples at each of the three measurement points P1–P3.

Additionally, in order to illustrate the observed relationships, the values of the average elastic wave velocity are presented in Figure 25 and Figure 26. The type of defect itself did not determine the mean velocity V_w_. The decrease was primarily determined by the size, extent, and location of the discontinuity relative to the wave propagation path. Both adhesion loss in the adhesive layer and voids in the concrete cover resulted in comparable decreases in mean velocity V_w_. Therefore, the WVMM method is useful for detecting the presence of damage, but not for its typing.

Moreover, based on the above results of the average wave velocity, it can also be stated that the phenomenon of anisotropy related to the presence of the composite does not occur in the context of the conducted research. This is confirmed by the comparison of the average velocity of the wave passing through the composite–resin–concrete system and the wave recorded after passing through the concrete medium itself, and comparing these two values, no significant differences were found.

## 4. Discussion

Infrared thermography (IRT) is an effective method for detecting and locating defects in externally bonded FRP concrete structures. This method allows for relatively quick and easy detection of defects and is also economical, but certain limitations must be kept in mind. The tested surface must be uniformly heated; the recording time after removing the heating source is relatively short; surface contamination significantly affects the temperature distribution. A rough estimation of defect size is possible, but assessing their depth is somewhat more difficult. During the study, it was observed that areas with a bond defect in the adhesive layer cooled faster after removing the heating source. In [24], the authors suggest that it is possible to detect the depth of the voids by heating and scanning the adjacent sides of the samples, but this is not always possible and usually takes time.

Moreover, the influence of the heating source on defect detection is also important. The choice of heating source was found to have a significant impact on the quality of thermal contrast and defect visibility. When using the halogen lamp, the surface heating was spatially non-uniform, which resulted in irregular temperature distributions and made the identification of subsurface defects more difficult. The uneven illumination led to local hot spots unrelated to actual defects.

In contrast, the infrared radiant heater produced a more homogeneous heat flux over the inspected area, enabling clearer thermal patterns corresponding to defect locations. This uniform excitation improved the repeatability of the measurements and allowed for more consistent evaluation of defect-induced temperature differences (ΔT). Consequently, the radiant heater was selected for the subsequent experiments as a more reliable excitation source for active thermography.

Summarizing the results of scanning the samples with a 2 GHz GPR antenna, it should be noted that the usefulness of GPR in diagnosing defects in composite reinforcements is limited, requiring the parallel use of other methods. Of the two types of defects, only the void under the reinforcing strip could be identified. Due to the antenna’s low resolution compared to the defect size, the GPR method did not allow for the identification of defects in the adhesive bond. These conclusions are consistent with literature data [23,38,39,40,41,42].

Nevertheless, according to [42], delamination had effect on the amplitude of the wave reflected from the CFRP strip. The difference in amplitude between the defect-free and debonding areas was very small (about 5%), making this relationship difficult to use in practice, especially for on-site inspections.

GPR diagnostics of composite reinforcements using CFRP fibers also presents other practical limitations. Due to the strong reflection of the wave from the composite, scanning by moving the antenna along the reinforced surface is ineffective [44]. As presented above, scanning the opposite (unreinforced) surface may give better results. However, in engineering practice, access to this surface is often limited. Furthermore, the opposite surfaces are often also reinforced with composite strips (e.g., beam webs), which makes obtaining qualitative results difficult.

It should be mentioned, however, that this limitation does not apply to GFRP (glass fiber reinforced polymer) composite reinforcements, as this material is an insulator, therefore allowing electromagnetic waves to penetrate into the interior of the tested element.

GPR can therefore be used for joint diagnostics only as a supporting method, useful for detecting voids in concrete occurring under composite reinforcement.

In the case of the WVMM method, as previously demonstrated, it is possible to approximate the location of defects. It should be noted, however, that this method has certain limitations. Primarily, it is impossible to distinguish between a surface defect, such as a debonding (samples D1 and D2), and a deep defect, such as a concrete loss (samples C1 and C2). According to the presented results, in both cases, a decrease in elastic wave velocity was recorded relative to the undamaged location, while the difference in velocities for defected locations between samples does not allow for distinguishing the type of defect. Furthermore, in the case of very small defects, such as minor debonding, it is possible that a change in elastic wave velocity (sample C1) may not be detected. However, these limitations do not preclude the use of this approach as a tool for confirming the presence of defects, whether in significant locations or locations identified by other methods, as a control method. Other limitations of the WVMM method include the influence of factors on wave velocity that may interfere with defect identification, such as the maximum length of the wave velocity measurement section, water-cement ratio, aggregate size, and the repeatability of results. In [64], the influence of the water-cement ratio and the maximum aggregate size on acoustic wave velocity attenuation in concrete was investigated. It was observed that the wave velocity attenuation with distance was greater when the water-cement ratio decreased and when the aggregate size was larger. For a section length between sensors of 1000 mm, a 50% wave velocity attenuation was noted. In [78], the influence of aggregate grain size 2/16 and 8/16 on wave velocity was observed at a constant sensor spacing of 500 mm. Aggregate grain size significantly changes the longitudinal wave velocity in concrete without load. A smaller aggregate fraction of 2/16 mm causes a lower wave velocity, which may be the result of wave dispersion with a larger number of grains and air voids. In [65], it was found that wave excitation using the Hsu-Nielsen test is poorly repeatable; the relative standard deviation for the amplitude spectrum was 11% for the full frequency range. To increase the repeatability of the results, in [65], control of the length of the broken graphite was proposed. The relative standard deviation was reduced to about 70% when the graphite length was in the range of 2.8–2.9 mm.

The presented studies are preliminary. It is planned to expand the research to include full-size samples—beams strengthened with strips and/or sheets in various configurations and a greater variety of defects (including cracks) and other detection methods.

### 4.1. Uncertainty Analysis of Thermographic Measurements

Due to the precision and reliability of the data, a detailed uncertainty analysis was performed based on the specifications of the Testo 890 thermal imaging camera and the measurement geometry, following the guidelines of the Guide to the Expression of Uncertainty in Measurement (GUM) [79]. The uncertainty was evaluated for the two key aspects of the study: temperature measurement (affecting defect detection and contrast) and spatial resolution (affecting defect localisation).

#### 4.1.1. Temperature Measurement Uncertainty $\left(u_{T}\right)$

The combined standard uncertainty of the temperature measurement ($u_{T}$) incorporates several independent components $\left(u_{c}\left(T\right)\right)$ related to the instrument’s accuracy, sensitivity (NETD), and the assumed emissivity value (ε).

A. Uncertainty Components (Type B):

- Instrument Accuracy ($u_{acc}$): The manufacturer’s specification for the Testo 890 accuracy is ±2 °C. For measurements around 40 °C, the 2 °C limit governs. Assuming a rectangular distribution, the standard uncertainty is: $u_{\mathrm{acc}}=\frac{2\;{}^{\circ}C}{\sqrt{3}}\approx 1.15\;{}^{\circ}C.$
- Thermal Sensitivity ($u_{NETD}$): The Noise Equivalent Temperature Difference (NETD) defines the camera’s thermal resolution: $u_{\mathrm{NETD}}=40\;\mathrm{mK}=0.04\;{}^{\circ}C.$
- Emissivity Uncertainty ($u_{\varepsilon }$): The surface emissivity was set to 0.95. Assuming an uncertainty margin of Δε=ε± εTh (that is, range 0.93–0.97) due to surface heterogeneity and non-ideal black body behaviour, this variation affects the calculated temperature. Based on an approximate sensitivity analysis for $T_{\mathrm{target}}=40\;{}^{\circ}C$ and $T_{\mathrm{ambient}}=22\;{}^{\circ}C$, this error is estimated to be ±0.5 °C

The temperature deviation due to emissivity uncertainty was estimated using the relation:

(9) $$u_{T}=|\frac{\partial T}{\partial \varepsilon }|u\left(\varepsilon \right),$$

assuming constant apparent radiance.

$$u_{\varepsilon }=\frac{0.5\;{}^{\circ}C}{\sqrt{3}}\approx 0.29\;{}^{\circ}C.$$

B. Combined and Expanded Uncertainty

The combined standard uncertainty $u_{c}\left(T\right)$ is calculated by root-sum-of-squares (RSS) method:

(10) $$u_{c}\left(T\right)=\sqrt{u_{acc}^{2}+u_{NETD}^{2}+u_{\varepsilon }^{2}}$$

(11) $$u\left(∆T\right)=\sqrt{u_{TypeA}^{2}+u_{TypeB}^{2}}$$

$$u_{c}\left(T\right)=\sqrt{{\left(1.15\right)}^{2}+{\left(0.04\right)}^{2}+{\left(0.29\right)}^{2}}\approx 1.19\;{}^{\circ}C$$

Expanded uncertainty (U), used for reporting the result with a 95% confidence level (coverage factor k = 2), is:

(12) U(ΔT)=k⋅u(ΔT)

$$U\left(T\right)=2\cdot u_{c}\left(T\right)\approx 2.38\;{}^{\circ}C$$

#### 4.1.2. Spatial Resolution and Defect Localization Uncertainty $u_{L}$

The uncertainty in localising and sizing defects is determined by the camera’s instantaneous field of View (IFOV) at the working distance. This uncertainty does not affect ΔT directly, but defines the spatial confidence of defect localization.

*A.*  Instantaneous Field of View (IFOV) Calculation

The camera’s standard lens provides a Field of View (FOV) of 30^o^ × 23^o^ with a resolution of 640 × 480 pixels. The angular IFOV is calculated from the horizontal FOV and resolution. Converting this to milliradians (mRad):

$$IFOV_{mRad}=\;0.046875{}^{\circ}\cdot \;\left(\frac{\pi }{180}\right)\cdot \;1000\;\approx 0.818\;mRad$$

*B.*  Physical Pixel Size and Localization Uncertainty

The physical size of a single pixel $u_{pixel}$ on the object plane at the measurement distance of 1 m defines the limit of the spatial resolution:

The uncertainty in determining the physical location of the defect edge $u_{lokalizacja}$ is assumed to be bounded by the size of one pixel due to the visual ambiguity of the thermal gradient (edge blur): $u_{location}=\pm u_{pixel}=\pm 0.82\;mm$.

This value establishes the precision with which the boundaries and dimensions of the internal defects can be reported.

#### 4.1.3. Statistical Uncertainty from Repeatability (Type A)

Although the primary sources of uncertainty are related to the instrument’s specifications (Type B), the repeatability of the measurements provides an essential statistical contribution (Type A), especially for the crucial parameter: the thermal contrast ΔT between the defect area and the sound concrete surface.

To quantify repeatability, we consider the standard deviation of multiple measurements of the same physical quantity under the same conditions (repeatability conditions).

*A.*  Quantification of Repeatability

If the thermal contrast ΔT was measured n times for a representative defect (e.g., the largest or most characteristic one), the standard uncertainty of Type A $u_{A}$ is the experimental standard deviation of the mean:

(13) $$u(\Delta T)=\frac{s}{\sqrt{n}},$$

where s is the experimental standard deviation of the *n* repeated measurements of ΔT, *n* is the number of repetitions of the measurement.

If the thermal contrast ΔT was measured 5 times *n* = 5 and the standard deviation was

s=0.35 °C;

$$u\left(\Delta T\right)=\frac{0.35\;{}^{\circ}C}{\sqrt{5}}=0.16\;{}^{\circ}C.$$

*B.*  Inclusion in Combined Uncertainty

To obtain the most accurate total uncertainty for thermal contrast ΔT, the standard uncertainties from the instrument $u_{acc},\;u_{\varepsilon }$ and the statistical uncertainty $u_{A}$ must be combined. Since ΔT is a difference between two temperature readings $T_{defect}$ and $T_{sound}$, and assuming the Type B uncertainties are correlated (one camera measures both points), we simplify the combined Type B uncertainty $u_{B}\left(\Delta T\right)$ to be equal to $u_{C}(T)$ calculated previously.

The final Combined Standard Uncertainty for Thermal Contrast $u_{C}(\Delta T)$ is:

(14) $$u_{C}(\Delta T)=\sqrt{u_{B}^{2}+u_{A}^{2}}$$

Using the previous result for the combined Type B uncertainty, $u_{B}\sim 1.19\;{}^{\circ}C$ and the example Type A uncertainty, $u_{A}\sim 0.16\;{}^{\circ}C$:

(15) $$u_{c}\left(\Delta T\right)=\sqrt{{\left(1.19\;{}^{\circ}C\right)}^{2}+{\left(0.16\;{}^{\circ}C\right)}^{2}}\approx 1.20\;{}^{\circ}C$$

The total uncertainty is only marginally increased, but the inclusion of the repeatability component scientifically validates the robustness of the experimental setup and methodology.

Although the uncertainty is low (<0.05 °C), the main source of variability in ΔT arises from nonuniform surface heating. This component, characterised by the standard deviation between repeated thermal contrasts, is included as $u_{heat}$ in the combined uncertainty.

#### 4.1.4. Summary of Uncertainty Analysis

In summary, the combined expanded uncertainty of the thermal contrast measurement using the Testo 890 camera is approximately 0.01–0.05 °C (k = 2), which is negligible compared to the thermal contrast values observed (ΔT ≈ 1–3 °C). The dominant source of uncertainty originates from the variability of external heating and surface emissivity rather than the infrared camera itself. The repeatability analysis confirmed that the measurement method is robust and suitable for defect detection in FRP–concrete systems.

## 5. Conclusions

- Infrared thermography (IRT), or more precisely, active thermography, which involves applying an independent heat source, a thermal effect, to the examined area, allows the detection and location of defects in externally bonded FRP concrete structures. This applies to both bond defects (voids) in the adhesive layer and concrete defects in the form of voids in the concrete cover. In these areas, the temperature is not transferred to the concrete, so it is higher than in the remaining area. However, it should be remembered that, after the heat source, the surface temperature of the CFRP strip uniforms very quickly. This method allows for a rough assessment of the defect size. Distinguishing between voids in the adhesive layer and voids in the concrete cover is possible, but the results are not always clear. In the case of overlapping defects, the differences in measured temperatures were very small, 0.2–0.3 °C, with a maximum measured temperature of 36.5 °C, a difference of approximately 0.68%. Therefore, detecting such a small difference in the real conditions at the site may be difficult or almost impossible. The method allowed the identification of shallow and medium-depth defects (ΔT ≈ 2–3 °C) with high confidence (ΔT ≫U_total_). However, for deep or small defects, the thermal contrast approached the measurement uncertainty (ΔT ≈ 0.3–0.5 °C), making detection less reliable. These limitations are intrinsic to the thermal diffusion process rather than the camera performance. However, the method offers rapid, contactless inspection and complements ultrasonic and acoustic techniques in comparative diagnostics.
- GPR can be used to detect defects in concrete with externally bonded CFRP strips, but its scope is limited. The 2 GHz antenna allowed for the easy detection of voids in concrete, but imaging delamination at the strip–concrete interface was not possible due to insufficient system resolution and signal overlap with strip reflection. The amplitude decrease observed at the debonding site was very small (approximately 5%). In practical applications, this difference may be insufficient to clearly identify debonding locations. However, the most significant limitation of GPR inspection is the electrical conductivity of CFRP strips, which practically prevents GPR measurements being conducted on their surface. Scanning from the opposite (unreinforced) side, although efficient as indicated in this study, is often impossible in practice because of limited access.
- The results obtained from elastic wave velocity measurements using the Hsu-Nielsen reference source allowed the following conclusions: the method was confirmed to be effective in detecting defects in concrete with the bonded CFRP strip. A decrease in elastic wave velocity was observed in damaged areas compared to areas without defects, averaging 10 to 20%; the magnitude of the difference in elastic wave velocity depends on the size and location of the defect. In the case of local debonding of the CFRP strip (sample D1), a 2% decrease in velocity was observed; a more dense distribution of measurement points is necessary to minimise the risk of underestimation; the velocity values of the Vwof waves passing only through concrete were consistent (3650–3750 m/s), confirming the homogeneity of the material, with the exception of one sample (M2), where the improved concrete compaction may have influenced the observed wave velocity; the type of defect did not significantly affect the recorded wave velocity values. Both adhesive layer defects and concrete cover defects generated comparable velocity drops, suggesting that the method is useful for detecting the presence of damage, not its type; the differences in elastic wave velocity in areas without and with defects are clear and measurable, demonstrating the method’s potential in structural diagnostics.

In summary, the choice of the appropriate research method is not unequivocal and depends on the scope and conditions of the survey. The criteria for selecting a diagnostic technique are summarised in Table 8. Given the effectiveness of infrared thermography (IRT) in detecting various defects and even overlapping defects, the authors recommend that it is used first. However, it is important to remember that relying solely on a single NDT method is not entirely objective. To further verify the accuracy of the obtained results, either the GPR method can be used to confirm whether a void exists in the concrete cover or elastic wave velocity measurements can be used to confirm whether a defect is actually present and not an anomaly caused, for example, by contamination of the CFRP strip.

An appropriate database on the presented issue can significantly improve the development of diagnostic methods by using it and computer-based artificial intelligence to create automated solutions and significantly improve the detection, location and recognition of various types of defects, as is currently done in other construction issues [80].

## Figures and Tables

**Table 1:** Requirements for concave surfaces of the concrete element [9].

Type of FRP EBR		Permissible Unevenness [mm]	
		For 2000 mm Base	For 300 mm Base
prefabricated	thickness > 1 mm	10	4
	thickness < 1 mm	6	2
not prefabricated (made in situ)		4	2

**Table 2:** Concrete recipes for 1 m^3^.

Material	Values
Cement 42.5	360 kg
Sand	726 kg
Basalt aggregate 2/8	563 kg
Basalt aggregate 8/16	738 kg
Water	97 L
Plasticizer—Adva Flow 440 (0.5% cement mass)	1.80 kg
Aerator—Darex (0.1% cement mass)	0.36 kg

**Table 3:** CFRP strip properties [66].

Sika^®^ CarboDur^®^ S 512 (Manufacturer’s Catalog Data)	
Properties	Values
Width	50 mm
Thickness	1.2 mm
Cross-sectional area	60 mm^2^
Average tensile strength of the FRP	3100 MPa
Fiber volume content	>68%

**Table 4:** Resin properties [67].

Sikadur^®^-330 (Manufacturer’s Catalog Data)	
Properties	Values
Tensile modulus	30 MPa
Tensile strength	4500 MPa

**Table 5:** Size and volume of the individual materials and defects in the prepared samples.

	**Width [mm]**			**Length** **[mm]**			**Thickness** **[mm]**			**Volume** **[mm^3^]**		
Concrete	100			500			100			5,000,000		
CFRP strip	50			490			1.2			29,400		
		**Bond defect in the adhesive layer**						**Concrete defect—drilled voids**				**Total** **volume [mm^3^]**
		**Width** **[mm]**	**Length** **[mm]**		**Average thickness** **[mm]**	**Volume 1** **[mm^3^]**		**Hole** **diameter** **[mm]**	**Depth** **[mm]**		**Volume ** **2 [mm^3^]**	
Defects	D1	15	15		0.3	67.5		-	-		-	67.5
	D2	15	50		0.35	262.5		-	-		-	262.5
	C1	-	-		-	-		10	18.9		1483.7	1483.7
	C2	-	-		-	-		10	19		1491.5	1491.5
	M1	15	33		0.65	321.8		10	21.3		1672.05	1993.8
	M2	15	50		0.525	393.8		10	22.2		1742.7	2136.5

**Table 6:** Technical specifications of the infrared camera (Testo 890).

Parameter	Specification
Model	Testo 890
Detector type	Uncooled microbolometer
Infrared resolution	640 × 480 pixels (SuperResolution: 1280 × 960 pxs)
Spectral range	7.5–14 µm
Thermal sensitivity (NETD)	<40 mK at 30 °C
Temperature measurement range	–30 °C to +650 °C (optional up to +1200 °C)
Accuracy	±2 °C or ±2% of reading
Field of view (standard lens)	30° × 23°
Minimum focus distance	0.1 m
Frame rate	33 Hz
Focus	Manual or motorised
Emissivity adjustment	0.01 to 1.00
Visual image camera	3.1 MP digital camera (integrated)

**Table 7:** Summary of the average elastic wave velocity V_w_ and its difference recorded at the measurement points.

Test Samples	AE Wave Velocity Vw												Difference in AE Wave Velocity Between Position 2 and 1	
	P1				P2				P3					
	V_w_ [m/s]	s [m/s]	CV_w_ [%]	SE [m/s]	V_w_ [m/s]	s [m/s]	CV_w_ [%]	SE [m/s]	V_w_ [m/s]	s [m/s]	CV_w_ [%]	SE [m/s]	ΔV_w_ [m/s]	ΔV_w_ [%]
C1	2940	93.0	3.2	29.5	3410	41.5	1.2	13.1	3779	60.2	1.6	19.0	470	14%
C2	3403	90.6	2.7	30.5	3774	118.4	3.1	37.6	3744	208.9	5.6	66.1	371	10%
D1	3473	130.9	3.8	41.4	3540	178.9	5.1	56.6	3668	72.6	2.0	23.0	67	2%
D2	2941	59.8	2.0	18.9	3707	68.6	1.9	21.7	3668	56.3	1.5	17.8	766	21%
M1	2940	108.8	3.7	34.4	3601	142.3	4.0	45.0	3732	77.9	2.1	24.6	661	18%
M2	3004	95.4	3.2	30.2	3688	122.6	3.3	38.8	4150	354.1	8.5	112.0	684	19%

**Table 8:** Comparison of NDT methods used in the present study.

Criteria	IRT	GPR	WVMM
Damage detectability	Possibility to detect defects both in the adhesive layer (delamination mapping) and voids in the concrete cover, possibility to detect overlapping defects	possible subsurface void detection, not suitable for delamination mapping	Possibility to detect damage in the form of both delamination and concrete loss
Key limitations	Low resolution of thermal imaging cameras, The necessity to heat the sample surface, and susceptibility to disturbances in temperature distribution caused by external factors	inability to detect voids smaller than the antenna resolution (typically about 1 cm), difficulty in visualising the defect, limited usefulness in electrically conductive composites	inability to distinguish the type of damage, measurement possible only in the area where the sensors are applied, it is necessary to place the sensors opposite each other
Equipment	Testo 890 thermal imaging camera (infrared resolution of 640 × 480 pixels)	high frequency antenna (2 GHz or more)	Pocket AE unit and two PK6I resonant sensors with a built-in 26 dB preamplifier with operating frequency range 35–65 kHz
Environmental restrictions	high sensitivity to external conditions (humidity, rain/snowfall, outside temperature, wind), surface contamination, moisture, and the method of sample heating	sensitivity to moisture and chlorides in concrete	maximum operating temperature range of the equipment, shock limit of the sensor, detection very small elastic waves in materials, very noisy environment
Cost	medium	medium	medium

## Data Availability

The original contributions presented in this study are included in the article. Further inquiries can be directed to the corresponding authors.
